# Idiopathic pulmonary fibrosis patients with severe physiologic impairment: characteristics and outcomes

**DOI:** 10.1186/s12931-020-01600-z

**Published:** 2021-01-06

**Authors:** Jean Pastre, Scott Barnett, Inga Ksovreli, Jeannie Taylor, A. Whitney Brown, Oksana A. Shlobin, Kareem Ahmad, Vikramjit Khangoora, Shambhu Aryal, Christopher S. King, Steven D. Nathan

**Affiliations:** 1 Inova Advanced Lung Disease and Transplant Program, Inova Fairfax Hospital, 3300 Gallows Road, Falls Church, VA 22042 USA; 2 Service de Pneumologie Et Soins Intensifs, Hôpital Européen Georges Pompidou, APHP, Paris, France

**Keywords:** Idiopathic pulmonary fibrosis, Pulmonary function test, 6-min walk test, Transplant-free survival, Hospitalization, Antifibrotic therapies

## Abstract

**Research question:**

There is no widely accepted grading system for IPF disease severity, although physiologic impairment based on pulmonary function testing is frequently employed. We sought to describe clinical and functional characteristics as well as outcomes of patients with severe physiologic impairment.

**Patients and methods:**

IPF patients with severe physiologic impairment defined by FVC ≤ 50% and/or DLco ≤ 30% predicted evaluated in the Inova Advanced Lung Disease Program between 2011 and 2019 were included. Demographic, physiologic, functional treatment and outcome data were collated.

**Results:**

There were 531 patients with IPF evaluated of whom 242 (46%) had severe physiologic impairment. Mean age was 72 ± 8 years; baseline FVC was 53 ± 17% and DL_CO_ 28 ± 9% of predicted. The mean 6 min walks test (6MWT) distance was 304 ± 121 m with 59% of the patients requiring supplemental oxygen (${\text{6MWT}}_{{{\text{O}}_{{2}} }}$ group). There was a poor correlation between the 6MWT distance and both FVC% and DLco%. Patients in the 6MWT_RA_ group had a better transplant-free survival than the ${\text{6MWT}}_{{{\text{O}}_{{2}} }}$ group (p = 0.002). Patients managed before October 2014 and not receiving antifibrotic therapy had worse outcomes with reduced transplant-free survival compared with patients presenting after this date who did receive antifibrotic therapy (n = 113) (log rank p < 0.0001).

**Conclusion:**

IPF patients often present with severe physiologic impairment which may be poorly correlated with their functional status. Assessment of IPF disease severity should not be based on physiologic impairment alone, but should also encompass functional status as well as need for supplemental oxygen. Antifibrotic therapy in patients with severe physiologic impairment is associated with improved outcomes.

## Background

Idiopathic pulmonary fibrosis (IPF) is a rare disease characterized by an irreversible, progressive loss of lung function [1–3]. Despite the desire for and advantages of an early diagnosis, a significant number of IPF patients have more advanced disease when initially diagnosed, while others evolve to having more severe disease. There is no widely accepted grading system for IPF, although physiologic impairment with a low forced vital capacity (FVC) and/or low diffusing capacity for carbon monoxide (DLco) are broadly used to characterize disease severity. However, these resting physiologic measurements are inexact in discerning the spectrum of IPF disease severity [4–6]. Indeed, mortality rates and the prevalence of complicating pulmonary hypertension have been shown to be equivalent across a wide spectrum of lung function impairment [4, 5].

IPF clinical trials of antifibrotic therapies have typically targeted patients with mild to moderate disease by employing cut points for the FVC of > 50% and the DLco of > 30–35% [7–9]. Therefore patients with a “severe” phenotype have been excluded from most clinical trials of antifibrotic therapy. Similarly, patients who initially present with milder impairment but progress to more advanced disease despite treatment with antifibrotic therapy are often not candidates for enrollment in add-on treatment trials. Despite some recent data in this population [10–13], there remains a large void of information regarding the efficacy of antifibrotic therapy in patients with severe disease.

We hypothesize that there is a wide spectrum of functional impairment in patients with severe disease based on their pulmonary function testing (PFT). We further hypothesize that these patients are good candidates and tolerant of antifibrotic therapy. We therefore sought to investigate the incidence, characteristics and outcomes of patients with severe physiologic impairment evaluated at a tertiary care ILD center.

## Methods

The records of all patients with IPF evaluated at the Inova Advanced Lung Disease Program, a tertiary referral center, for the period January 2011 to February 2019 were reviewed. Patients were diagnosed based on prior international consensus guidelines for the diagnosis of IPF [14, 15]. Patients qualified for study inclusion if they presented with or developed severe physiologic impairment as defined by a FVC ≤ 50% predicted and/or a DLco ≤ 30% predicted. It is standard practice at our center for all new consults to undergo both pulmonary function testing (PFTs) and a 6 min walk test (6MWT) during the initial consultation. Data from the first visit of patients who presented with severe physiologic impairment was collated. For those patients who developed severe impairment during follow-up, data was collated at the time that they breached one of the two severe thresholds. Information recorded included demographics: age, gender, height, weight, body mass index (BMI), smoking status and date of initial consultation, as well as PFT and 6MWT data. PFT parameters recorded included the FVC, FVC% predicted, forced expiratory volume in first second (FEV_1_), FEV_1_% predicted, FEV_1_/FVC ratio, DLco, DLco% predicted, total lung capacity (TLC) and TLC% predicted. PFTs were performed according to the American Thoracic Society (ATS) standards [16, 17] (*V6200 Autobox DL; SensorMedics, Yorba Linda, CA*) and expressed as percent prediction using the predicted equations of NHANES for lung volumes and Crapo for the DLco [18, 19]. The 6MWT was performed according to the ATS guidelines with the instruction to walk as far as possible in 6 min using standard verbal prompts [20]. Patients were walked on room air (RA). If during the course of the walk they desaturated below 80% for 15 s or more, then the walk test was halted. If their oxygen saturation (SpO_2_) dropped below 88% during the RA walk, then a second walk was performed on supplemental oxygen titrated to maintain a SpO_2_ > 88%. We report the results from the RA walk test if their SpO_2_ remained > 86% (6MWT_RA_ group). Otherwise, data from the 6MWT performed with supplemental oxygen (${\text{6MWT}}_{{{\text{O}}_{{2}} }}$ group) was collated. 6MWT distance (6MWD), oxygen saturation, heart rate and Borg score were measured and recorded at baseline and at 6 min. Antifibrotic treatment use and tolerance was recorded.

Outcome data including respiratory-related hospitalization, lung transplantation, or death were obtained from the electronic medical record, the Inova Advanced Lung Disease database, and the Social Security death index. Hospitalization events captured included only respiratory-related non-elective admissions of at least 24 h in duration. Analyses of patient outcomes included transplant-free survival and a composite endpoint of time to first respiratory-related hospitalization, death or lung transplantation. We also analyzed each of these components of this composite endpoint separately. A further outcome analysis of those who presented with severe disease to those who developed severe disease was undertaken. Time zero for the outcome analyses was the date of the PFTs that qualified the patients as having severe physiologic impairment. All dates of last follow-up, hospitalization, death, or lung transplantation were recorded.

A number of outcome analyses were undertaken which included a subgroup analysis of the 6MWT_RA_ versus ${\text{6MWT}}_{{{\text{O}}_{{2}} }}$ populations. Patients were also divided between those not treated and managed before October 2014 (date of FDA approval for both nintedanib and pirfenidone), and those treated with either agent after this date or with early access to either of these drugs. To qualify for the latter group, treatment needed to have been prescribed within 1 month after the PFTs documenting severe physiologic impairment. This ensured that patients who were started on treatment before developing severe physiologic impairment were excluded from all outcome analyses of antifibrotic therapy. An additional outcome analysis compared those patients initially evaluated before October 2014 to those seen after this date, irrespective of antifibrotic therapy. This analysis was undertaken to negate any selection bias associated with the implementation of antifibrotic therapy. Local IRB committee approval for this study was obtained before any data collection or analyses (U19-08-3702).

### Statistical considerations and data analysis

All demographic and pulmonary function data are presented as the mean ± standard deviation (SD) or the median, depending on the distribution. Group comparisons were performed using *Student’s* t-test or *Wilcoxon’s* rank sum test for continuous variables; while *Pearson’s* chi-square test or *Fisher’s* exact test were used for categorical variables, where appropriate.

The Spearman correlations between the 6MWT distance and PFT parameters were analyzed. *Kaplan–Meier* survival analyses and the log-rank test were used to compare transplant-free survivals and time to first respiratory-related hospitalization, death or transplantation. Lastly, we utilized Cox Proportion hazards models to determine the association of survival time with selected parameters (e.g., age, gender BMI, etc.). Hazard ratios (HR) and 95% confidence limits (CL) are presented. Both univariate and multivariate Cox models are presented to demonstrate both the effects of individual parameter on survival time and subsequent effects following adjustment for all other modeled parameters. All statistical analyses were performed using GraphPad (*GraphPad*^*®*^*; ver. 7, La Jolla, CA*) and SAS (SAS^®^; *ver. 9.4, Cary, NC*) with p-values ≤ 0.05 considered statistically significant.

## Results

There were 531 patients with IPF evaluated at our center during the period of the study, 185 (35%) of whom met the inclusion criteria of physiologic impairment with FVC ≤ 50% and/or a DLco ≤ 30% predicted at their first presentation, and a further 57 (11%) progressing and breaching at least one of these thresholds during follow-up. Both FVC and DLco data were available for 192 of the total severe patient population (n = 242). The DLco was not available for the remaining 50 others, due to the patients inability to perform this procedure. Mean FVC was 53 ± 17% and mean DLco was 28 ± 9% predicted. 95 patients (39%) were included based on FVC ≤ 50% predicted, 94 (39%) based on DLco ≤ 30% predicted and 53 (22%) for both FVC and DLco under those cut points. Demographic and physiologic characteristics of the study population are shown in Table 1. Additional file 1: Table S1 shows the baseline characteristics of the study population divided between those who presented initially with severe disease (n = 185) and those who progressed to develop severe disease (n = 58). With regards to associated comorbidities, there were 51 patients (21%) with severe obstructive sleep apnea, 5 patients presented with or developed a lung cancer. There were 99 patients (41%) with evidence of elevated pulmonary arterial pressure on transthoracic echocardiography. There were 80 patients (33%) who underwent a right heart catheterization with 43 (54%) having a mean pulmonary arterial pressure ≥ 25 mmHg.

**Table 1:** Demographics, physiologic and functional data of all patients with severe functional impairment (FVC ≤ 50% and/or DLco ≤ 30% predicted)

	All patients	FVC ≤ 50% predicted only	DLco ≤ 30% predicted only	Both FVC ≤ 50% and DLco ≤ 30% predicted	p-value
Patients, n (%)	242	95 (39%)	94 (39%)	53 (22%)	
Male, n (%)	196 (81%)	74 (78%)	79 (84%)	43 (81%)	0.08
Age, years ± SD	72 ± 8	72 ± 8^†^	73 ± 8*^§^	71 ± 7^†^	0.01
BMI, mean ± SD	28 ± 5	27 ± 5	27 ± 5	31 ± 6	0.15
Smokers, n, % (mean PY ± SD)	155, 68% (30 ± 23)	54, 60%^†^ (22 ± 20)	69, 73%* (34 ± 25)	32, 60% (32 ± 21)	0.02
FVC% ± SD	53 ± 17	42 ± 7	70 ± 17	43 ± 5	
DLco% ± SD	28 ± 9	39 ± 9	24 ± 4	24 ± 6	
Available 6MWT, n (%)	231 (95%)	91 (99%)	92 (98%)	48 (91%)	
6MWD, meters ± SD	304 ± 121	309 ± 123	319 ± 117^§^	266 ± 116^†^	0.04
${\text{6MWT}}_{{{\text{O}}_{{2}} }}$, n (%)	140 (59%)	50 (54%)	53 (56%)	37 (70%)	0.08
${\text{6MWT}}_{{{\text{O}}_{{2}} }}$ mean flow rate, L/min	5	4	5	5	
6MWT SpO_2_ nadir, % ± SD	88 ± 5	89 ± 5	88 ± 6	88 ± 5	0.51
6MWT max Borg, mean ± SD	4 ± 2	4 ± 2	4 ± 2	4 ± 2	0.29

BMI: body mass index; SD: standard deviation; PY: pack-year; FVC%: forced vital capacity, % predicted; DLco%: single breath diffusing capacity for carbon monoxide, % predicted; 6MWT: 6-min walk test; ${\text{6MWT}}_{{{\text{O}}_{{2}} }}$: patients with need for supplemental oxygen during 6MWT; 6MWD: 6-min walk test distance; SpO_2_: blood oxygen saturation

^*^ p value < 0.05 when compared to patients with FVC ≤ 50% predicted only

^†^ p value < 0.05 when compared to patients with DLco ≤ 30% predicted only

^§^ p value < 0.05 when compared to patients with both FVC ≤ 50% and DLco ≤ 30% predicted

Concomitant 6MWT data was available in 231 of the patients (95%). Mean distance was 304 ± 121 m with 59% of the patients in the ${\text{6MWT}}_{{{\text{O}}_{{2}} }}$ group (mean flow 5 L/min) (Table 1). Patient characteristics, PFT and 6MWT data categorized by the two 6MWT groups are shown in Table 2. Those patients in the ${\text{6MWT}}_{{{\text{O}}_{{2}} }}$ group had shorter walks (281 ± 118 vs 337 ± 115 m respectively, p = 0.0005), more pronounced desaturation (despite supplemental oxygen) (86 ± 5 vs. 91 ± 5% respectively, p < 0.0001) and higher Borg scores (5 ± 2 vs. 3 ± 2 respectively, p = 0.0008) than those in the 6MWT_RA_ group. Interestingly, there was no difference in the FVC% between the two groups, but the DLco% was lower in the ${\text{6MWT}}_{{{\text{O}}_{{2}} }}$ group.

**Table 2:** Clinical, physiological and functional characteristics of IPF patients with severe functional impairment (FVC ≤ 50% and/or DLco ≤ 30% predicted) based on their exercise need for supplemental oxygen (6MWT_RA_ versus ${\text{6MWT}}_{{{\text{O}}_{{2}} }}$)

	All patients	6MWT_RA_	${\text{6MWT}}_{{{\text{O}}_{{2}} }}$	p-value
Patients, n (%)	242	95/231 (41%)	136/231 (59%)	
Male, n (%)	196 (81%)	78 (82%)	113 (83%)	0.86
Age, years ± SD	72 ± 8	74 ± 8	71 ± 8	0.003
BMI, mean ± SD	28 ± 5	26 ± 4	29 ± 6	0.0004
Smokers, n, % (mean PY ± SD)	155, 68% (30 ± 23)	58, 61% (25 ± 20)	93, 68% (32 ± 25)	0.26
FVC% ± SD	53 ± 17	54 ± 16	52 ± 19	0.51
DLco% ± SD	28 ± 9	30 ± 9	26 ± 9	0.02
Mean O_2_ flow rate, L/min		0	5	
6MWD, meters ± SD	304 ± 121	337 ± 115	281 ± 118	0.0005
6MWT SpO_2_ nadir, % ± SD	88 ± 5	91 ± 5	86 ± 5	< 0.0001
6MWT Borg max, mean ± SD	4 ± 2	3 ± 2	5 ± 2	0.0008

6MWT: 6-min walk test; 6MWT_RA_: patients able to complete a 6MWT with SpO_2_ > 86% on room air; ${\text{6MWT}}_{{{\text{O}}_{{2}} }}$: patients with need for supplemental oxygen during 6MWT; SD: standard deviation; BMI: body mass index; PY: pack-year; FVC%: forced vital capacity, % predicted; DLco%: single breath diffusing capacity for carbon monoxide; % predicted; 6MWD: 6-min walk test distance; SpO_2_: blood oxygen saturation

**Fig. 1:**
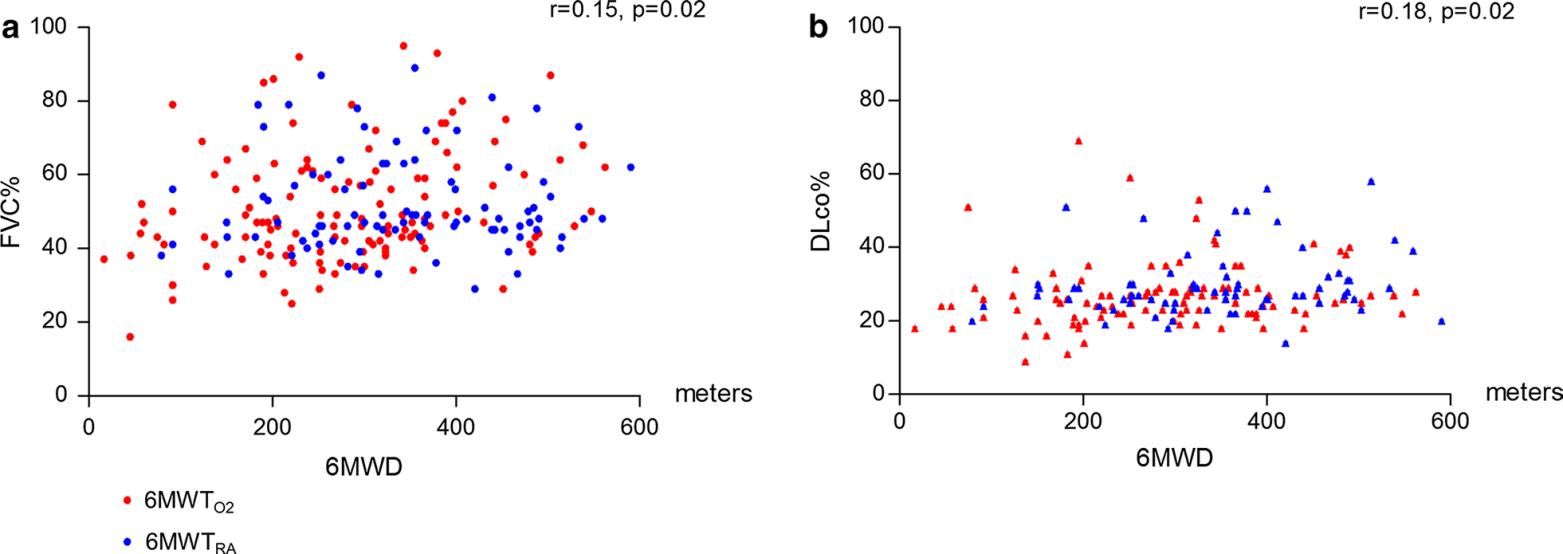
Correlation between 6-min walk test distance (6MWD) and FVC% (**a**) or DLco% (**b**) for all IPF patients diagnosed with severe functional impairment (FVC ≤ 50% and/or DLco ≤ 30% predicted) based on the need for supplemental oxygen at exercise (${\text{6MWT}}_{{{\text{O}}_{{2}} }}$, red) or not (6MWT_RA_, blue). 6MWD: 6-min walk test distance, meters; FVC%: forced vital capacity, % predicted; DLco%: single breath diffusing capacity for carbon monoxide, % predicted; 6MWT_RA_: patients able to complete a 6MWT with SpO_2_ > 86% on room air; ${\text{6MWT}}_{{{\text{O}}_{{2}} }}$: patients with need for supplemental oxygen during 6MWT

Table 3 provides an analysis of the 6MWT categorized by various cut points and combinations of disease severity based on the FVC% and DLco%. Based on these PFT categories, there was a wide range of 6MWT distances observed. At the one end of the spectrum, patients with a FVC ≤ 50% but a relatively preserved DLCO > 35%, had a 6MWD of 366 m ± 120; while at the other end of the spectrum, those patients with a FVC ≤ 40% in association with a DLco ≤ 25% had an average 6MWD of only 219 m ± 120. We observed no correlation between the 6-min walk test distance and both FVC% and DLco% (r = 0.15, p = 0.02 and r = 0.18, p = 0.02 respectively, Fig. 1).

**Table 3:** Patient distribution based on pulmonary function testing and associated 6-min walk test (6MWT) data

	All FVC%	FVC ≤ 50%	FVC ≤ 45%	FVC ≤ 40%	FVC > 50%
All DLco%					
n	242	147	92	45	95
6MWD, m	304 ± 121	294 ± 123	275 ± 124	260 ± 120	318 ± 116
${\text{6MWT}}_{{{\text{O}}_{{2}} }}$	61%	60%	65%	70%	61%
DLco > 35%					
n	24	24	14	5	0
6MWD, m	366 ± 120	366 ± 120	356 ± 131	333 ± 138	–
${\text{6MWT}}_{{{\text{O}}_{{2}} }}$	50%	50%	64%	60%	–
DLco ≤ 35%					
n	168	73	45	21	95
6MWD, m	301 ± 119	277 ± 136	258 ± 123	264 ± 104	318 ± 116
${\text{6MWT}}_{{{\text{O}}_{{2}} }}$	63%	67%	67%	76%	61%
DLco ≤ 30%					
n	151	57	34	15	95
6MWD, m	298 ± 119	262 ± 117	246 ± 122	237 ± 102	318 ± 116
${\text{6MWT}}_{{{\text{O}}_{{2}} }}$	64%	71%	67%	87%	61%
DLco ≤ 25%					
n	81	32	22	10	49
6MWD, m	281 ± 123	253 ± 127	226 ± 119	219 ± 120	297 ± 119
${\text{6MWT}}_{{{\text{O}}_{{2}} }}$	74%	74%	68%	80%	74%
DLco ≤ 20%					
n	31	16	12	7	15
6MWD, m	246 ± 128	234 ± 114	237 ± 128	234 ± 125	257 ± 145
${\text{6MWT}}_{{{\text{O}}_{{2}} }}$	77%	77%	70%	86%	77%
DLco not available					
n	50	50	33	19	0
6MWD, m	281 ± 118	281 ± 118	261 ± 112	224 ± 81	–
${\text{6MWT}}_{{{\text{O}}_{{2}} }}$	57%	57%	65%	79%	–

PFT: pulmonary function test; FVC: forced vital capacity, % predicted; DLco: single breath diffusing capacity for carbon monoxide, % predicted; 6MWT; 6-min walk test; 6MWD: 6-min walk test distance, meters ± SD; ${\text{6MWT}}_{{{\text{O}}_{{2}} }}$: patients with need for supplemental oxygen during 6MWT

The median duration of follow-up was 18 ± 15 months (range 0–80 months) with 27 patients being lost to follow-up. Of the remaining 215 severe patients, 136 (63%) required a respiratory-related hospitalization with 52 (38%) admitted for acute exacerbations, 49 patients (23%) were transplanted while 79 (37%) died. The major reasons for not listing patients for lung transplantation included advanced age (46%), comorbidities/general condition (43%), while 8 patients refused and 7 were considered “too well” for transplantation.

**Fig. 2:**
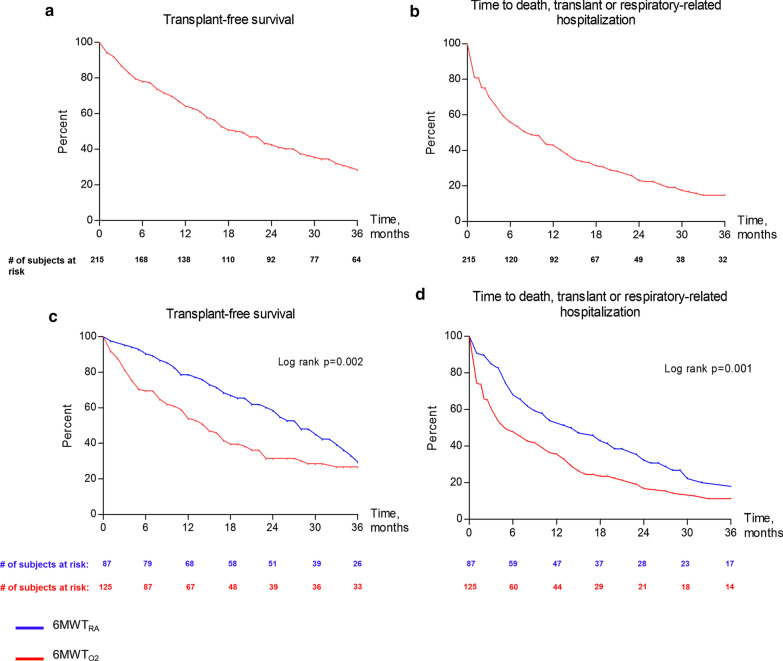
Kaplan–Meier curves of transplant-free survival, and time to first respiratory-related hospitalization, death or transplantation in IPF patients diagnosed with severe functional impairment (FVC ≤ 50% and/or DLco ≤ 30% predicted) (**a**, **b**). Kaplan–Meier curves of transplant-free survival stratified by need for supplemental oxygen during the 6 min walk test (${\text{6MWT}}_{{{\text{O}}_{{2}} }}$: red curve vs blue curve: 6MWT_RA_, **c**, **d**). FVC: forced vital capacity, % predicted; DLco: single breath diffusing capacity for carbon monoxide, % predicted; 6MWT: 6-min walk test; 6MWT_RA_: patients able to complete a 6MWT with SpO_2_ > 86% on room air; ${\text{6MWT}}_{{{\text{O}}_{{2}} }}$: patients with need for supplemental oxygen during 6MWT

Kaplan–Meier analyses of transplant-free survival and time to first respiratory-related hospitalization, death or transplantation are shown in Fig. 2a, b, respectively. Patients in the 6MWT_RA_ group had a better transplant-free survival and a longer time before first respiratory-related hospitalization, death or transplantation than those in the ${\text{6MWT}}_{{{\text{O}}_{{2}} }}$ group (log rank p = 0.002 and p = 0.001 respectively, Fig. 2c, d). We then looked at each component of this composite endpoint separately through Kaplan–Meier analyses of survival, survival with exclusion of transplanted patients and time to first respiratory-related hospitalization (Additional file 2: Figure S1a–c respectively). Patients who underwent a lung transplant were censored as alive on the date of transplantation for both the hospitalization and survival analyses. Additional file 3: Figure S2 demonstrates a survival comparison of those who presented with severe disease to those who developed severe disease.

**Fig. 3:**
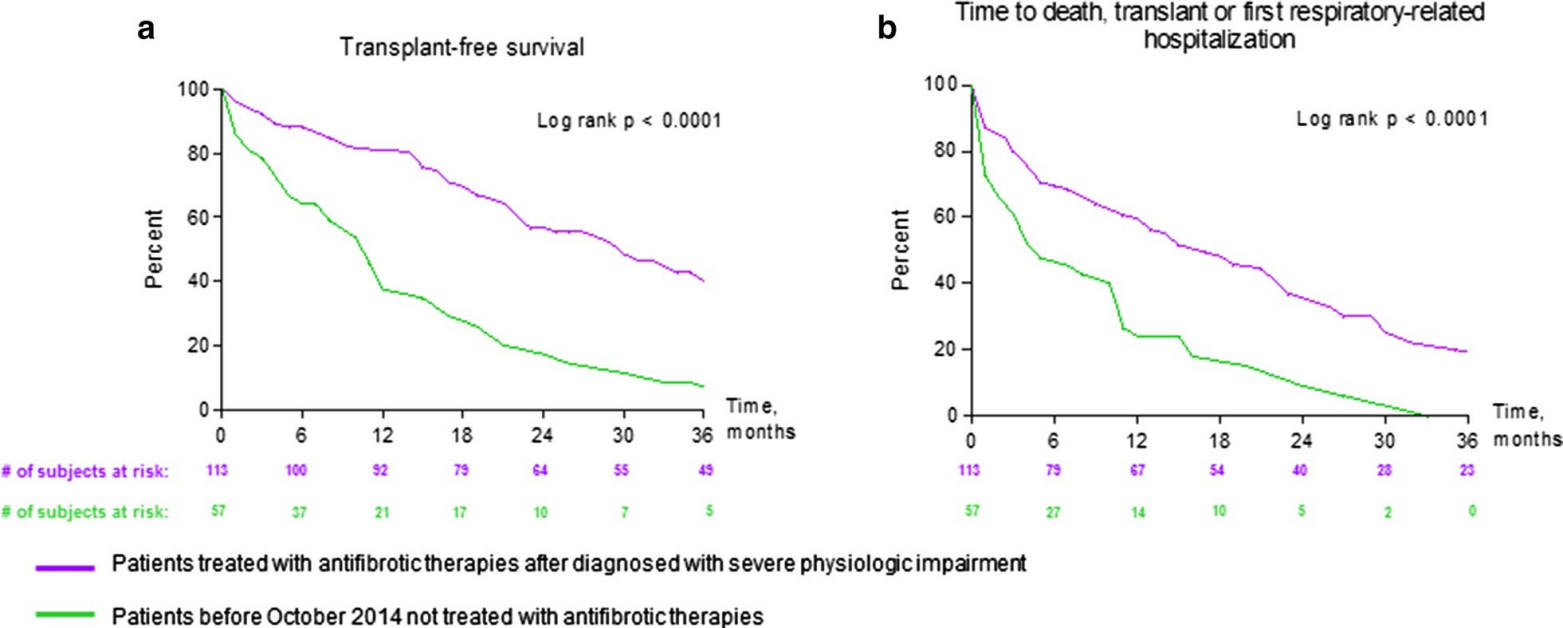
Kaplan–Meier curves of transplant-free survival (**a**), and time to first respiratory-related hospitalization, death or transplantation (**b**) in IPF patients diagnosed with severe functional impairment (FVC ≤ 50% and/or DLco ≤ 30% predicted) stratified by treatment with antifibrotic therapies; n = 115 patients (purple), versus n = 57 patients (green) presenting prior to October 2014 and who were not treated

Of the 242 patients, 163 were placed on antifibrotic therapy with 113 patients (67%) placed on antifibrotic at their initial evaluation (mean FVC%: 56 ± 19%, mean DLco%: 29 ± 9%) (Additional file 4: Figure S3). Fifty seven patients were untreated as they were evaluated prior to October 2014. The remaining 22 patients were not started on treatment for a variety of reasons including patient refusal (n = 1), ongoing cigarette smoking (n = 1), social issues (n = 1) and transition to hospice care (n = 19) (Additional file 4: Figure S3). Drug distribution of the 113 treated patients was as follows: 61 were treated with pirfenidone (54%), 32 with nintedanib (28%) and 20 had both sequentially (18%). Mean duration of treatment was 16 ± 14 months (range 0–53 months). Fifty nine patients (52%) experienced side effects which included gastrointestinal upset (77%), loss of appetite or asthenia (29%) and cutaneous manifestations (15%). In 38 patients (34%), dose reduction was necessary, while 26 (23%) required discontinuation of therapy. Kaplan–Meier curves of transplant-free survival and time to first respiratory-related hospitalization, death or transplantation stratified by treatment with antifibrotic therapies are shown in Fig. 3a, b. Patients managed before October 2014 and not receiving one of the two antifibrotic agents through early access had distinctly worse outcomes with a lower transplant-free survival and a shorter time before first respiratory-related hospitalization, death or transplantation than those receiving either drug prescribed after the development of severe physiologic impairment (log rank p < 0.0001). We performed a further outcome analysis of patients managed prior to October 2014 versus those managed after this date, so as to account for any bias of the 22 non-treated patients in the latter group (Additional file 5: Figure S4a, b). This demonstrated similar results with better outcome for those patients presenting after October 2014.

Bivariate and multivariate analyses of transplant-free survival accounting for the following variables was performed: age, gender, history of smoking, body mass index (BMI), FVC%, DLco%, need for supplemental oxygen during the 6MWT, 6MWD and treatment with antifibrotic therapy (Table 4). Multivariate analysis identified male gender (p = 0.008) and FVC% below the median (≤ 47% predicted) (p = 0.01) to be associated with worse transplant-free survival, whereas treatment with antifibrotic therapies was associated with a better prognosis (p = 0.003).

**Table 4:** Multivariate Cox-proportional hazards ratios and 95% confidence intervals for transplant-free survival

Parameter	Univariate			Multivariate		
	HR	95% CL	p	HR	95% CL	p
Age, per 5 years	0.88	0.77–1.00	0.056	0.90	0.76–1.06	0.21
Male	1.64	0.96–2.79	0.068	2.37	1.26–4.46	0.008
Hx of smoking	0.97	0.66–1.42	0.871	0.93	0.57–1.53	0.07
BMI	0.99	0.95–1.03	0.721	0.95	0.90–1.01	0.08
FVC ≥ 47%	0.66	0.46–0.94	0.023	0.57	0.37–0.88	0.01
DLco ≥ 27%	0.89	0.62–1.29	0.540	0.84	0.54–1.29	0.42
Oxygen use	1.72	1.19–2.49	0.004	1.55	0.99–2.43	0.058
6MWD, per 50 m	0.95	0.86–1.05	0.335	0.95	0.85–1.08	0.48
Antifibrotic therapies	0.48	0.32–0.71	< 0.001	0.51	0.33–0.80	0.003

HR: hazard ratio; CL: confidence limit; BMI: body mass index; FVC%: forced vital capacity, % predicted; DLco%: single breath diffusing capacity for carbon monoxide, % predicted; FVC% and DLco% stratified by median (FVC: 47%; DLco: 28%); 6MWD: 6-min walk test distance, meters

## Discussion

IPF patients with FVCs ≤ 50% of predicted and/ or DLco ≤ 30% of predicted have typically been regarded as having severe disease. The fact that almost one third of IPF patients presenting to our tertiary care center for the first time met one or both of these criteria underscores that there are still inordinate delays in both diagnosis and referral. Patients with this degree of physiologic impairment have been excluded from clinical trials of antifibrotic therapies. Therefore there is a dearth of information about the clinical course, functional impairment as well as the safety and efficacy of antifibrotic therapy in this population, although mining of registry data or recent real-world papers have focused on this issue, providing encouraging results in this neglected population [10–13, 21].

Our study is the first to provide insight into the functional ability and oxygen needs of IPF patients with severe physiologic impairment. We describe a wide range in distances walked highlighting the discordance between severe physiologic and functional impairment. The fact that 41% of these “severe” patients did not require supplemental oxygen to complete their 6MWTs was somewhat of a surprise which further emphasizes this discordance. Our findings challenge the common belief that patients with severe physiological disease have similarly severe functional impairment. We therefore believe that functional ability and need for supplemental oxygen need to be accounted for and taken into consideration when assessing disease severity. The GAP index (gender, age, physiology) is the most commonly accepted prognostic scoring system with others such as the composite physiologic index (CPI) also in existence, but neither of these includes a measure of functional ability or need for supplemental oxygen [22, 23]. Our analysis reinforces that any future scoring system of disease severity should encompass these parameters in order to more accurately depict the impact of the disease on both function and outcomes.

Historically, IPF patients with severe physiologic impairment have been excluded from clinical trials of antifibrotic therapy based on the belief that their efficacy may only be demonstrable in those with mild to moderate disease, rather than in severe patients for whom “the horse might be out of the barn”. Our analysis, however, provides a rationale to include patients with more severe physiological impairment in future clinical trials. As clinical trial designs venture beyond the FVC for future endpoints, the issue of what endpoints to use and how these might perform is raised. We demonstrate through our outcomes analysis that IPF patients with severe physiologic impairment are at high risk for other meaningful events including hospitalization, need for lung transplantation and death. Inclusion of these patients will cast a wider net and not only enable more robust and expeditious recruitment, but with a high event rate could also render shorter trials more feasible.

Our outcome analysis demonstrated a strong association between antifibrotic use and survival. In order to ensure a homogenous group of patients with severe disease, we specifically excluded patients who were already on antifibrotic therapy when they developed severe physiologic impairment so that patients who had mild to moderate impairment at the initiation of treatment did not bias our outcomes results. The difference in transplant-free survival became apparent early and appeared profound. For example, at 6 and 12 months transplant-free survival in the treatment arm was 11% and 17% versus 36% and 62% in the treatment naïve group, respectively. In addition, only 23% of our severe patients did not tolerate antifibrotic therapy, which is consistent with what we and other have reported previously for patients with more mild to moderate disease [24]. Our analysis supports the notion that a response to antifibrotic therapy is potentially demonstrable in clinical trials of severe IPF patients. More meaningful endpoints, such as hospitalization and death, can also be employed rather than the FVC, which appears more suited to a population who have more lung function to lose. One potential option for future IPF clinical trial inclusion is to forego a lower limit for the FVC% and DLco%, but to rather limit recruiting the most severe patients based on oxygen needs and 6MWT distance criteria.

There are a number of limitations to our study. The high proportion of patients with severe disease presenting for the first time may be biased by the fact that we are a transplant center and some physicians may have referred patients when sufficiently severe to warrant transplant consideration. However, this is counter to the International Society for Heart and Lung Transplantation who recommend that IPF patients be referred for transplant consideration at the time of their diagnosis [25]. Our center is also well-recognized as an ILD center with multiple clinical trials, so we believe that this potential bias had a small impact on a “skew” to more severe patients. We did not evaluate patients’ computed tomographic scans of the chest to measure concordance between fibrosis scores, the presence of any emphysema and degree of physiologic impairment. It is quite possible that some of the patients with severe functional impairment may have had a lesser parenchymal lung disease burden, especially those who did not require supplemental oxygen. Our choice to stratify patients based as a room air 6MWT if their SpO_2_ remained > 86% is somewhat arbitrary, but there is no established SpO_2_ exercise nadir in IPF that has been shown to warrant the implementation of supplemental oxygen. This cut point was decided prior to any data analysis. Our survival analysis comparing those patients treated with antifibrotic versus not has inherent associated selection and historical biases. However, our demonstration of a survival benefit is consistent with prior reports including a subgroup analysis from the pivotal pirfenidone phase 3 studies of patients with the most severe disease in the context of those studies [26]. Our analysis differs, however, as our study population had even more physiologic impairment with equivalent numbers of patients qualifying as severe based on FVC% and DLco%. One additional limitation is that we did not have DLco data in all the patients, due to their inability to perform the maneuver. There were disproportionately more patients in the early cohort who met the outcome endpoints of lung transplantation rather than death. The reason for this disparity is uncertain, but could have magnified the outcome difference. A possible reason includes changes in the lung allocation score system; however, if anything this should have resulted in a bias toward more transplants in the most recent era. Liberalization of recipient criteria, such as increased age, should also have resulted in more transplants in the most recent era. Differences in wait times between eras are also unlikely as the average wait time at our program has consistently been around 3 months. Accreditation of our center as a Pulmonary Fibrosis Foundation center of excellence has resulted in many more referrals for ILD of elderly, non-transplant candidates over time. In addition, the availability of antifibrotic therapy has driven more referrals of patients who were previously told there was nothing that could be done and that they were not lung transplant candidates. One remaining conceivable explanation is that the implementation of antifibrotic therapy might have resulted in a delay or even avoided the need for transplant in some patients. Lastly, only a small proportion of our patients were evaluated at variable time intervals for pulmonary hypertension. Therefore we were unable to correlate the presence of pulmonary hypertension with the 6MWT, oxygen requirements, or patient outcomes; however, our group has reported on this previously [5, 27].

## Conclusion

In summary, we have provided unique insight into a group of patients with severe physiologic impairment and have shown that not all these patients have concordant severe functional compromise. We believe our analysis lays the foundation for a future scoring system that incorporates not only physiology, but also functional ability and supplemental oxygen requirement. We also hope that this report will embolden investigators and clinical trialists to include more severe patients in future clinical trials of antifibrotic therapy. It is only through a more inclusive approach that future therapies will be made available for this subgroup of IPF patients, who currently lack, and are in most dire need of, therapeutic options.

## Supplementary Information

**Additional file 1:** **Table S1.** Clinical, physiological and functional characteristics of IPF patients depending on when they develop severe functional impairment (FVC ≤ 50% and/or DLco ≤ 30% predicted). **Table S2.** Clinical, physiological and functional characteristics of IPF patients depending on whether they are treated or not by antifibrotic therapies.

**Additional file 2:** **Figure S1.** Kaplan–Meier curves of survival (a), survival but with exclusion of transplanted patients (b) and time to first respiratory-related hospitalization (c) in IPF patients diagnosed with severe functional impairment (FVC ≤ 50% and/or DLco ≤ 30% predicted). Abbreviation: LT: lung transplantation.

**Additional file 3:** **Figure S2.** Kaplan–Meier curves of transplant-free survival (a), and time to first respiratory-related hospitalization, death or transplantation (b) in IPF patients diagnosed with severe functional impairment (FVC ≤ 50% and/or DLco ≤ 30% predicted) stratified by those who presented with severe functional impairment at first consultation in our facility versus those who evolved toward it.

**Additional file 4:** **Figure S3.** Repartition of our population of IPF patients diagnosed with severe functional impairment (FVC ≤ 50% and/or DLco ≤ 30% predicted) regarding use of antifibrotic therapies.

**Additional file 5:** **Figure S4.** Kaplan-Meier curves of transplant-free survival (a), and time to first respiratory-related hospitalization, death or transplantation (b) in IPF patients diagnosed with severe functional impairment (FVC ≤ 50% and/or DLco ≤ 30% predicted) stratified by treatment with antifibrotic therapies: n = 137 patients, 115 patients with addition of the 22 non-treated patients in the group managed after October 2014 in order to account for any bias (purple), versus n = 57 patients (green) seen prior to October 2014 and who were not treated.

## Data Availability

The datasets used and analyzed during this study are available from the corresponding author on reasonable request.

## Abbreviations

6MWT: 6-Min walk test
6MWD: 6-Min walk test distance
6MWT_O2_: Patients with need for supplemental oxygen during 6MWT
6MWT_RA_: Patients without need for supplemental oxygen during 6MWT
ATS: American Thoracic Society
BMI: Body mass index
DLco: Single breath diffusing capacity for carbon monoxide
FDA: Food and Drug Administration
FVC: Forced vital capacity
ILD: Interstitial lung disease
IPF: Idiopathic pulmonary fibrosis
IRB: Institutional review board
O_2_: Oxygen
PFT: Pulmonary function test
PY: Pack-year
SD: Standard deviation
SpO_2_: Blood oxygen saturation

## References

[1] Ley B, Collard HR, King TE (2011). Clinical course and prediction of survival in idiopathic pulmonary fibrosis. Am J Respir Crit Care Med.

[2] Nathan SD, Shlobin OA, Weir N, Ahmad S, Kaldjob JM, Battle E (2011). Long-term course and prognosis of idiopathic pulmonary fibrosis in the new millennium. Chest.

[3] Richeldi L, Collard HR, Jones MG (2017). Idiopathic pulmonary fibrosis. Lancet Lond Engl.

[4] King TE, Safrin S, Starko KM, Brown KK, Noble PW, Raghu G (2005). Analyses of efficacy end points in a controlled trial of interferon-gamma1b for idiopathic pulmonary fibrosis. Chest.

[5] Nathan SD, Shlobin OA, Ahmad S, Urbanek S, Barnett SD (2007). Pulmonary hypertension and pulmonary function testing in idiopathic pulmonary fibrosis. Chest.

[6] Robbie H, Daccord C, Chua F, Devaraj A (2017). Evaluating disease severity in idiopathic pulmonary fibrosis. Eur Respir Rev.

[7] Noble PW, Albera C, Bradford WZ, Costabel U, Glassberg MK, Kardatzke D (2011). Pirfenidone in patients with idiopathic pulmonary fibrosis (CAPACITY): two randomised trials. Lancet Lond Engl.

[8] King TE, Bradford WZ, Castro-Bernardini S, Fagan EA, Glaspole I, Glassberg MK (2014). A phase 3 trial of pirfenidone in patients with idiopathic pulmonary fibrosis. N Engl J Med.

[9] Richeldi L, du Bois RM, Raghu G, Azuma A, Brown KK, Costabel U (2014). Efficacy and safety of nintedanib in idiopathic pulmonary fibrosis. N Engl J Med.

[10] Richeldi L, Kolb M, Jouneau S, Wuyts WA, Schinzel B, Stowasser S (2020). Efficacy and safety of nintedanib in patients with advanced idiopathic pulmonary fibrosis. BMC Pulm Med.

[11] Chung MP, Park MS, Oh IJ, Lee HB, Kim YW, Park JS (2020). Safety and efficacy of pirfenidone in advanced idiopathic pulmonary fibrosis: a nationwide post-marketing surveillance study in Korean patients. Adv Ther.

[12] Costabel U, Albera C, Glassberg MK, Lancaster LH, Wuyts WA, Petzinger U (2019). Effect of pirfenidone in patients with more advanced idiopathic pulmonary fibrosis. Respir Res.

[13] Vietri L, Cameli P, Perruzza M, Cekorja B, Bergantini L, d’Alessandro M (2020). Pirfenidone in idiopathic pulmonary fibrosis: real-life experience in the referral centre of Siena. Ther Adv Respir Dis.

[14] Raghu G, Collard HR, Egan JJ, Martinez FJ, Behr J, Brown KK (2011). An official ATS/ERS/JRS/ALAT statement: idiopathic pulmonary fibrosis: evidence-based guidelines for diagnosis and management. Am J Respir Crit Care Med.

[15] Raghu G, Remy-Jardin M, Myers JL, Richeldi L, Ryerson CJ, Lederer DJ (2018). Diagnosis of idiopathic pulmonary fibrosis. An official ATS/ERS/JRS/ALAT clinical practice guideline. Am J Respir Crit Care Med.

[16] Standardization of Spirometry, 1994 Update. American Thoracic Society. Am J Respir Crit Care Med. 1995;152(3):1107–36. 10.1164/ajrccm.152.3.7663792 7663792

[17] American Thoracic Society (1995). Single-breath carbon monoxide diffusing capacity (transfer factor). Recommendations for a standard technique—1995 update. Am J Respir Crit Care Med.

[18] Hankinson JL, Odencrantz JR, Fedan KB (1999). Spirometric reference values from a sample of the general U.S. population. Am J Respir Crit Care Med.

[19] Crapo RO, Morris AH (1981). Standardized single breath normal values for carbon monoxide diffusing capacity. Am Rev Respir Dis.

[20] Holland AE, Spruit MA, Troosters T, Puhan MA, Pepin V, Saey D (2014). An official European Respiratory Society/American Thoracic Society technical standard: field walking tests in chronic respiratory disease. Eur Respir J.

[21] O’Brien EC, Hellkamp AS, Neely ML, Swaminathan A, Bender S, Snyder LD (2020). Disease severity and quality of life in patients with idiopathic pulmonary fibrosis: a cross-sectional analysis of the IPF-PRO registry. Chest.

[22] Ley B, Ryerson CJ, Vittinghoff E, Ryu JH, Tomassetti S, Lee JS (2012). A multidimensional index and staging system for idiopathic pulmonary fibrosis. Ann Intern Med.

[23] Wells AU, Desai SR, Rubens MB, Goh NSL, Cramer D, Nicholson AG (2003). Idiopathic pulmonary fibrosis: a composite physiologic index derived from disease extent observed by computed tomography. Am J Respir Crit Care Med.

[24] Flaherty KR, Fell CD, Huggins JT, Nunes H, Sussman R, Valenzuela C (2018). Safety of nintedanib added to pirfenidone treatment for idiopathic pulmonary fibrosis. Eur Respir J.

[25] Weill D, Benden C, Corris PA, Dark JH, Davis RD, Keshavjee S (2015). A consensus document for the selection of lung transplant candidates: 2014—an update from the Pulmonary Transplantation Council of the International Society for Heart and Lung Transplantation. J Heart Lung Transplant.

[26] Nathan SD, Costabel U, Albera C, Behr J, Wuyts WA, Kirchgaessler K-U (2019). Pirfenidone in patients with idiopathic pulmonary fibrosis and more advanced lung function impairment. Respir Med.

[27] Lettieri CJ, Nathan SD, Barnett SD, Ahmad S, Shorr AF (2006). Prevalence and outcomes of pulmonary arterial hypertension in advanced idiopathic pulmonary fibrosis. Chest.

