# Supplementary material for: Idiopathic pulmonary fibrosis patients with severe physiologic impairment: characteristics and outcomes
Source: Respir Res. 2021 Jan 6;22:5. doi: 10.1186/s12931-020-01600-z (PMC7788925; doi:10.1186/s12931-020-01600-z)
Supplement: Supplementary file 1 — Additional file 1: Table S1. Clinical, physiological and functional characteristics of IPF patients depending on when they develop severe functional impairment (FVC ≤ 50% and/or DLco ≤ 30% predicted). Table S2. Clinical, physiological and functional characteristics of IPF patients depending on whether they are treated or not by antifibrotic therapies. [file 12931_2020_1600_MOESM1_ESM.docx]

Additional data

Additional Table S1: Clinical, physiological and functional characteristics of IPF patients depending on when they develop severe functional impairment (FVC ≤ 50% and/or DLco ≤ 30% predicted)

|  | **All patients** | **Severe physiological impairment at first presentation** | **Evolution towards severe physiological impairment** | **p-value** |
| --- | --- | --- | --- | --- |
| Patients, n (%) | 242 | 185 (76%) | 57 (24%) |  |
| Male, n (%) | 196 (81%) | 149 (81%) | 47 (82%) | 0.84 |
| Age, years ± SD | 72 ± 8 | 71 ± 8 | 71 ± 8 | 0.32 |
| BMI, mean ± SD | 28 ± 5 | 28 ± 5 | 29 ± 5 | 0.34 |
| Smokers, n, % (mean PY ± SD) | 155, 68%  (30 ± 23) | 118, 68%  (32 ±23) | 37, 67%  (26 ± 24) | > 0.9 |
| FVC% ± SD | 53 ± 17 | 52 ± 18 | 55 ± 15 | 0.28 |
| DLco% ± SD | 28 ± 9 | 28 ± 9 | 29 ± 10 | 0.50 |
|  |  |  |  |  |
| Available 6MWT, n (%) | 231 (95%) | 174 (94%) | 57 (100%) |  |
| 6MWD, meters ± SD | 304 ± 121 | 293 ± 122 | 336 ± 107 | 0.01 |
| 6MWT_O2_, n (%) | 140 (59%) | 109 (61%) | 31 (54%) | 0.44 |
| 6MWT_O2_ mean flow rate, L/min | 5 | 5 | 5 | 0.51 |
| 6MWT SpO_2_ nadir, % ± SD | 88 ± 5 | 88 ±5 | 89 ± 6 | 0.54 |
| 6MWT max Borg, mean ± SD | 4 ± 2 | 4 ± 2 | 4 ± 2 | 0.45 |

**Severe physiological impairment at first presentation**: patients who presented with severe physiologic impairment as defined by a FVC ≤ 50% predicted and/or a DLco ≤ 30% predicted at first consultation in our facility**; Evolution towards severe physiological impairment**: patients who developed severe physiologic impairment as defined by a FVC ≤ 50% predicted and/or a DLco ≤ 30% predicted during follow-up in our facility.

BMI: body mass index; SD: standard deviation; PY: pack-year; FVC%: forced vital capacity, % predicted; DLco%: single breath diffusing capacity for carbon monoxide, % predicted; 6MWT: 6-minute walk test; 6MWT_O2_: patients with need for supplemental oxygen during 6MWT; 6MWD: 6-minute walk test distance; SpO_2_: blood oxygen saturation

Additional Table S2: Clinical, physiological and functional characteristics of IPF patients depending on whether they are treated or not by antifibrotic therapies

|  | **All patients** | **Treated with antifibrotics** | **Not treated for antifibrotics** | **p-value** |
| --- | --- | --- | --- | --- |
| Patients, n (%) | 242 | 163 | 79 |  |
| Male, n (%) | 196 (81%) | 135 (83%) | 61 (77%) | 0.30 |
| Age, years ± SD | 72 ± 8 | 72 ± 8 | 68 ± 9 | 0.0004 |
| BMI, mean ± SD | 28 ± 5 | 28 ± 5 | 28 ± 5 | 0.65 |
| Smokers, n, % (mean PY ± SD) | 155, 68%  (30 ± 23) | 107, 66%  29 ± 23 | 48, 70%  33 ± 25 | 0.48 |
| FVC% ± SD | 53 ± 17 | 56 ± 19 | 47 ± 14 | 0.0005 |
| DLco% ± SD | 28 ± 9 | 28 ± 9 | 27 ± 10 | 0.23 |
|  |  |  |  |  |
| Available 6MWT, n (%) | 231 (95%) | 160 (98%) | 71 (90%) |  |
| 6MWD, meters ± SD | 304 ± 121 | 311 ± 116 | 289 ± 129 | 0.30 |
| 6MWT_O2_, n (%) | 140 (59%) | 86 (54%) | 54 (76%) | 0.001 |
| 6MWT_O2_ mean flow rate, L/min | 5 | 5 | 5 | 0.30 |
| 6MWT SpO_2_ nadir, % ± SD | 88 ± 5 | 89 ± 5 | 88 ± 5 | 0.28 |
| 6MWT max Borg, mean ± SD | 4 ± 2 | 4 ± 2 | 4 ± 2 | 0.80 |

BMI: body mass index; SD: standard deviation; PY: pack-year; FVC%: forced vital capacity, % predicted; DLco%: single breath diffusing capacity for carbon monoxide, % predicted; 6MWT: 6-minute walk test; 6MWT_O2_: patients with need for supplemental oxygen during 6MWT; 6MWD: 6-minute walk test distance; SpO_2_: blood oxygen saturation

Additional Figure S1: Kaplan-Meier curves of survival (Additional Figure S1 a), survival but with exclusion of transplanted patients (Additional Figure S1 b) and time to first respiratory-related hospitalization (Additional Figure S1 c) in IPF patients diagnosed with severe functional impairment (FVC ≤ 50% and/or DLco ≤ 30% predicted)

Abbreviation: LT: lung transplantation

Additional Figure S2: Kaplan-Meier curves of transplant-free survival (Additional Figure S2 a), and time to first respiratory-related hospitalization, death or transplantation (Additional Figure S2 b) in IPF patients diagnosed with severe functional impairment (FVC ≤ 50% and/or DLco ≤ 30% predicted) stratified by those who presented with severe functional impairment at first consultation in our facility versus those who evolved toward it

Additional Figure S3: Repartition of our population of IPF patients diagnosed with severe functional impairment (FVC ≤ 50% and/or DLco ≤ 30% predicted) regarding use of antifibrotic therapies
