# Supplementary figures and images for: Idiopathic pulmonary fibrosis patients with severe physiologic impairment: characteristics and outcomes
Source: Respir Res. 2021 Jan 6;22:5. doi: 10.1186/s12931-020-01600-z (PMC7788925; doi:10.1186/s12931-020-01600-z)

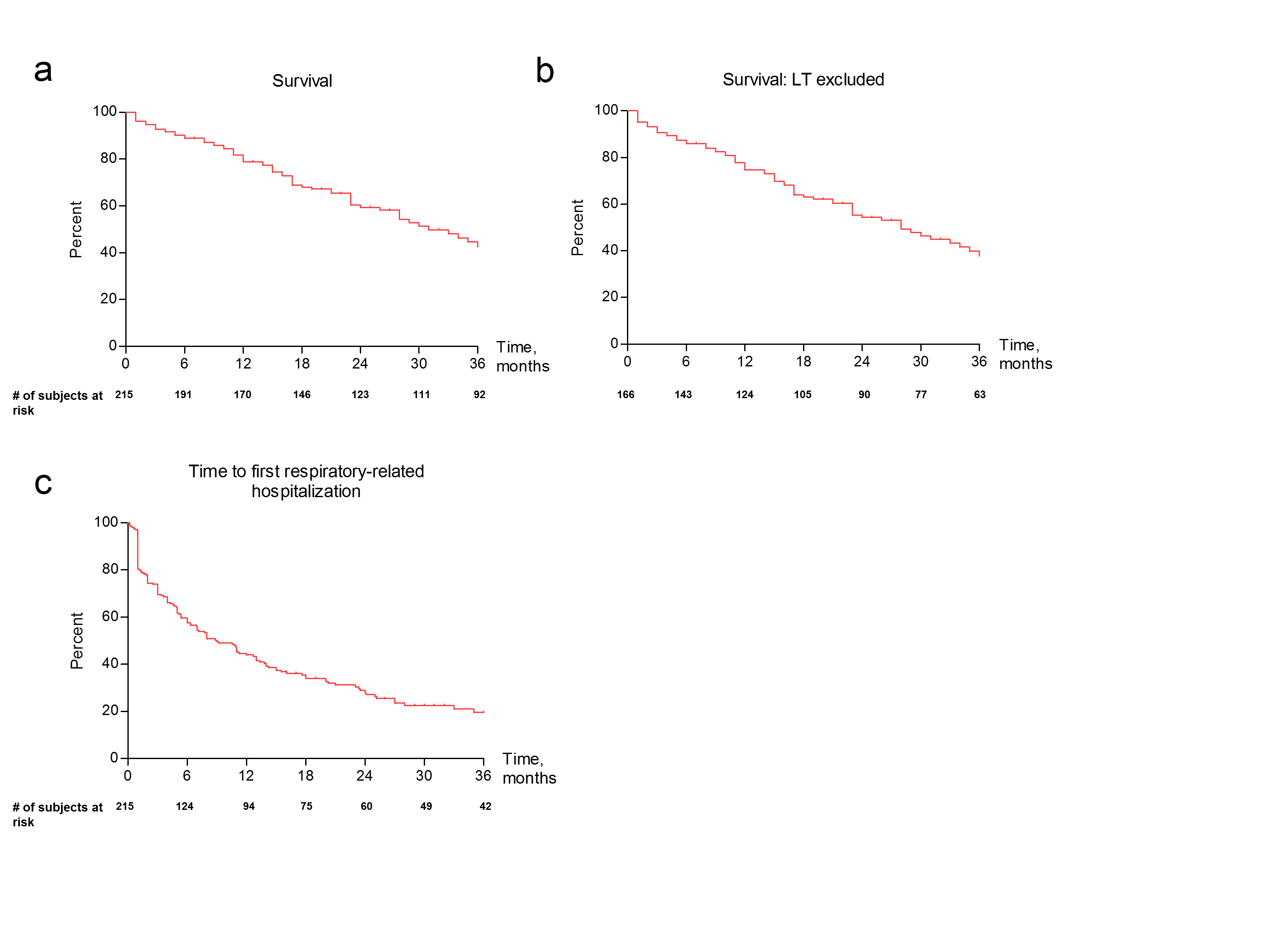

Supplement: Supplementary file 2 — Additional file 2: Figure S1. Kaplan–Meier curves of survival (a), survival but with exclusion of transplanted patients (b) and time to first respiratory-related hospitalization (c) in IPF patients diagnosed with severe functional impairment (FVC ≤ 50% and/or DLco ≤ 30% predicted). Abbreviation: LT: lung transplantation. [file 12931_2020_1600_MOESM2_ESM.tif]

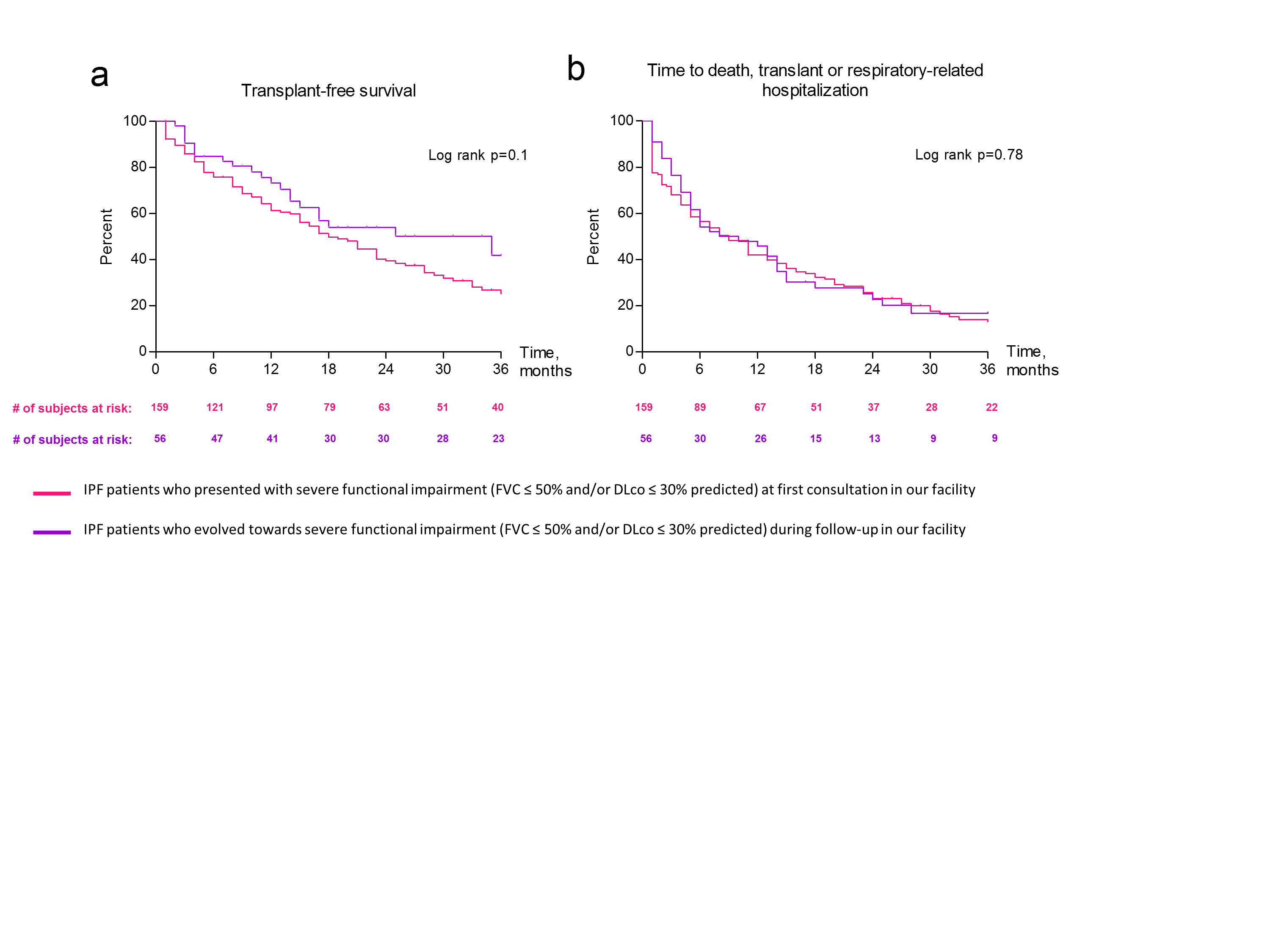

Supplement: Supplementary file 3 — Additional file 3: Figure S2. Kaplan–Meier curves of transplant-free survival (a), and time to first respiratory-related hospitalization, death or transplantation (b) in IPF patients diagnosed with severe functional impairment (FVC ≤ 50% and/or DLco ≤ 30% predicted) stratified by those who presented with severe functional impairment at first consultation in our facility versus those who evolved toward it. [file 12931_2020_1600_MOESM3_ESM.tif]

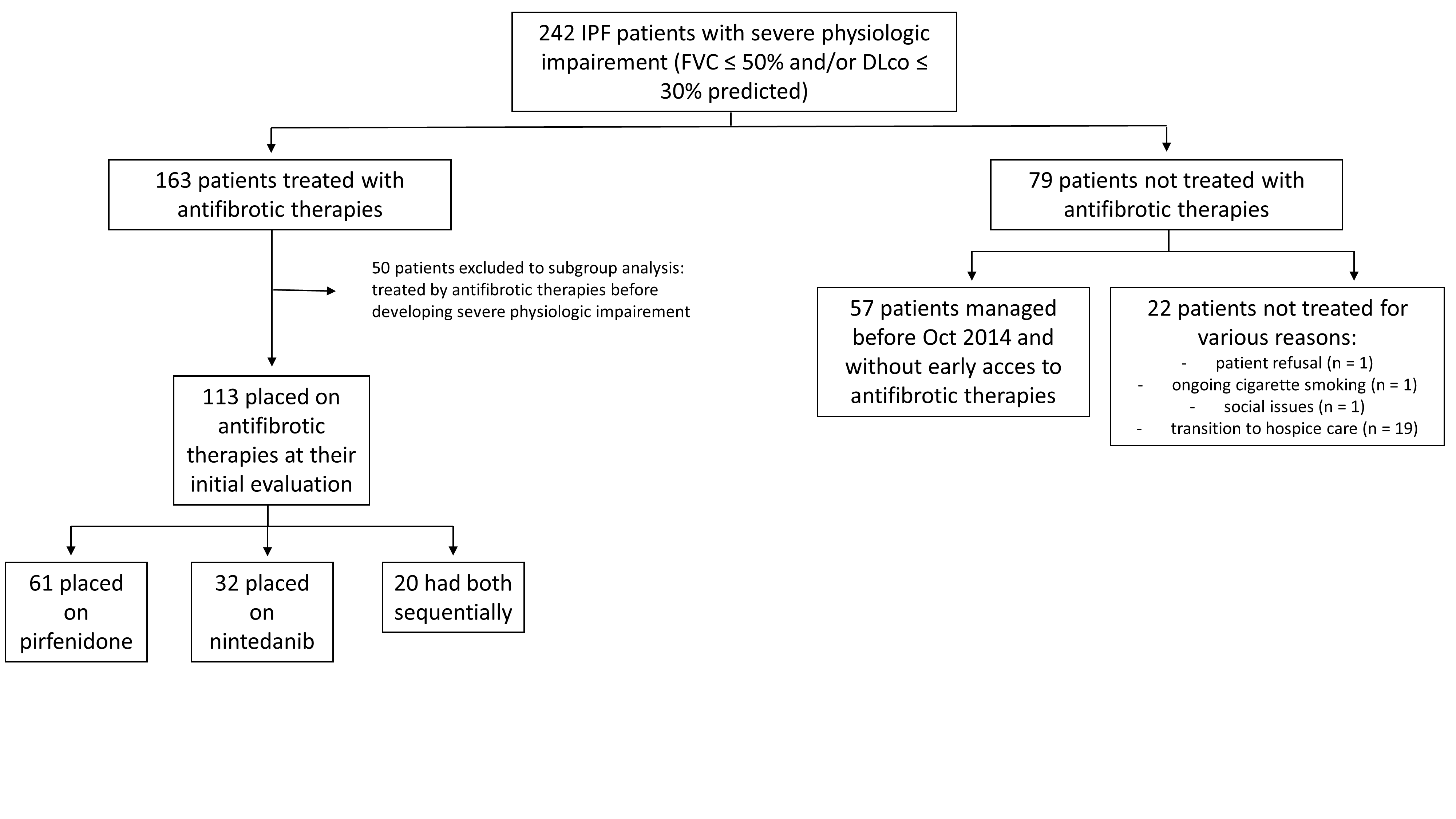

Supplement: Supplementary file 4 — Additional file 4: Figure S3. Repartition of our population of IPF patients diagnosed with severe functional impairment (FVC ≤ 50% and/or DLco ≤ 30% predicted) regarding use of antifibrotic therapies. [file 12931_2020_1600_MOESM4_ESM.tif]

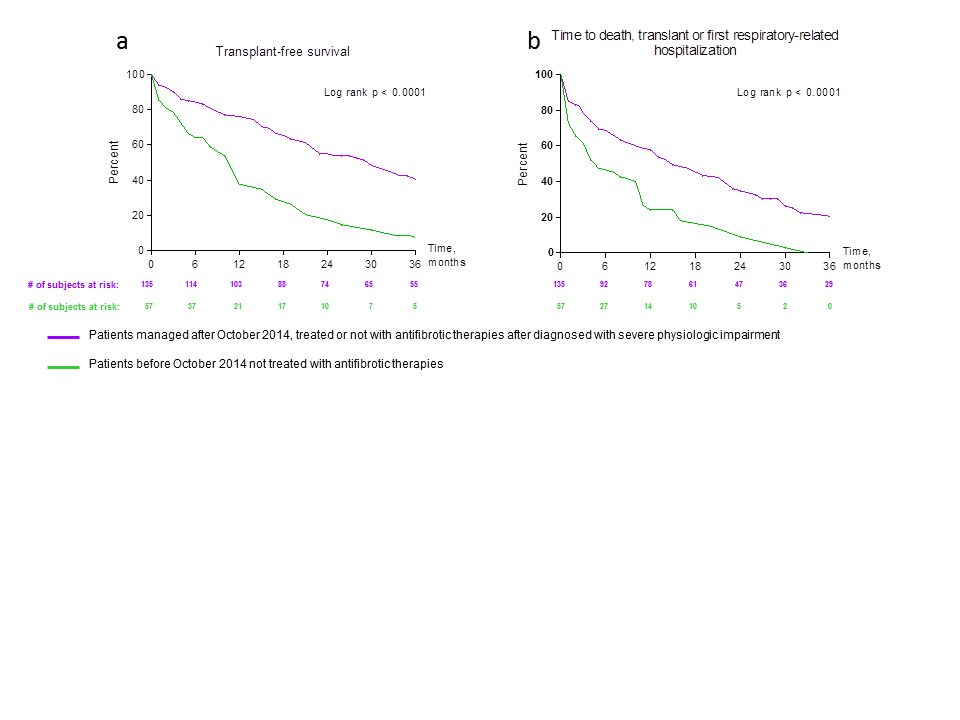

Supplement: Supplementary file 5 — Additional file 5: Figure S4. Kaplan-Meier curves of transplant-free survival (a), and time to first respiratory-related hospitalization, death or transplantation (b) in IPF patients diagnosed with severe functional impairment (FVC ≤ 50% and/or DLco ≤ 30% predicted) stratified by treatment with antifibrotic therapies: n = 137 patients, 115 patients with addition of the 22 non-treated patients in the group managed after October 2014 in order to account for any bias (purple), versus n = 57 patients (green) seen prior to October 2014 and who were not treated. [file 12931_2020_1600_MOESM5_ESM.tif]
